# A novel risk classification score for malignant ureteral obstruction: a multicenter prospective validation study

**DOI:** 10.1038/s41598-021-84054-7

**Published:** 2021-02-24

**Authors:** Kouji Izumi, Takashi Shima, Kazuyoshi Shigehara, Kiyoshi Sawada, Renato Naito, Yuki Kato, Mitsuo Ofude, Hiroshi Kano, Hiroaki Iwamoto, Hiroshi Yaegashi, Kazufumi Nakashima, Masashi Iijima, Shohei Kawaguchi, Takahiro Nohara, Yoshifumi Kadono, Atsushi Mizokami

**Affiliations:** 1 Department of Integrative Cancer Therapy and Urology, Kanazawa University Graduate School of Medical Science, 13-1 Takara-machi, Kanazawa, Ishikawa 920-8641 Japan; 2 Department of Urology, Toyama Prefectural Central Hospital, Toyama, Japan; 3 Department of Urology, Ishikawa Prefectural Central Hospital, Kanazawa, Japan; 4 Department of Urology, Municipal Tsuruga Hospital, Tsuruga, Japan; 5 Department of Urology, Komatsu Municipal Hospital, Komatsu, Japan; 6 Department of Urology, Fukui-Ken Saiseikai Hospital, Fukui, Japan; 7 Department of Urology, Kouseiren Takaoka Hospital, Takaoka, Japan; 8 Department of Urology, National Hospital Organization Kanazawa Medical Center, Kanazawa, Japan

**Keywords:** Cancer, Cancer therapy

## Abstract

Emergence of malignant ureteral obstruction (MUO) has been reported as a sign of poor prognosis; however, the distribution of survival time in patients with MUO is considerably wide, and no risk classification score has been constructed. To evaluate whether a novel risk classification score for overall survival that we previously developed, is effective in a large cohort. Investigator-initiated, prospective, multicenter diagnostic/prognostic study was conducted. Patients with MUO were divided into three risk groups based on the score calculated using four prognostic factors (PLaCT: Primary site, Laterality, serum Creatinine level, and Treatment for primary site) at the first visit, and prospective follow-up was performed. Overall survival and ureteral stent failure-free survival of each risk group were compared. In total, 300 patients with 21 different primary sites were enrolled. The numbers of patients in good, intermediate, and poor risk groups were 105, 106, and 89, respectively. Median survival times of patients in good, intermediate, and poor risk groups were 406, 221, and 77 days, respectively (*P* < 0.0001). In 217 patients with ureteral stenting, median ureteral stent failure-free survival times of good, intermediate, and poor risk groups were 385, 183, and 57 days, respectively (*P* < 0.0001). Limitations include the limited ethnicity and the extended duration of study enrollment. The novel PLaCT risk classification score could divide MUO patients into three risk groups with distinct survival times and ureteral stent patencies. This score will aid in establishing prognosis and treatment strategy for all physicians engaged in cancer treatment.

## Introduction

Malignant ureteral obstruction (MUO) is caused by diverse intrinsic and extrinsic lesions^1,2^. The appearance of MUO is regarded as a sign of poor prognosis; however, the distribution of survival times in patients with MUO is considerably wide because of heterogeneity among affected patients’ backgrounds^3,4^. A prognostic risk classification score for these patients is urgently needed in clinical practice. We previously conducted a retrospective study to develop a novel risk classification for MUO patients and clearly showed that our classification could divide MUO patients into three groups that significantly differed with respect to overall survival (OS), based on a score calculated using four factors which was named PLaCT risk classification score in the current study^5^. In addition, we clarified that gynecological (GY) cancer contributed to extended ureteral stent patency when used as management for MUO^5^. However, this risk classification was not reliable, and factors that contribute to ureteral stent patency remain uncertain because our prior study was retrospective and used a small sample of patients from a single institution. Therefore, this risk classification should be validated using a different cohort to increase the strength of the evidence, such that it can be applied to MUO patients in clinical practice. In the current study, we prospectively validated our risk classification score in a large sample of patients with MUO.

## Patients and methods

### Patients and data collection

The PLaCT study was an investigator-initiated, prospective, multicenter diagnostic/prognostic study that was conducted in accordance with the Declaration of Helsinki 1975, as revised in 2013. Advanced cancer patients with MUO who consulted urology departments at Kanazawa University Hospital and seven related hospitals were enrolled between February 1, 2013, and December 31, 2017. Patients who were diagnosed with malignant neoplasia based on histopathology or clinical course were eligible, irrespective of primary cancer site, stage, and age. All treatments and data collection for MUO were undertaken following written informed consent prior to registration. The treatment for MUO was determined at discretion of urologists in charge. Before treatment for MUO, the following clinical data were recorded for each patient: age, sex, Eastern Cooperative Oncology Group Performance Status (ECOG PS), primary cancer site, hematological and blood biochemical measurements including renal function [e.g., serum creatinine (sCr), blood urea nitrogen (BUN), and estimated glomerular filtration rate (eGFR)], and information regarding the treatment schedule for primary cancer. The location and length of MUO were also recorded, and the obstruction level was defined as upper, middle, or lower ureter, according to location above, over, or below the sacroiliac joint. In patients who exhibited different obstruction levels in bilateral ureters, the more severely obstructed side and length was selected for analysis. Finally, the type of urinary diversion (ureteral stent or nephrostomy) was recorded for each patient. The PLaCT study received approval from the Medical Ethics Committee of Kanazawa University, and the trial was registered in the University hospital Medical Information Network, Center identifier UMIN 000010335.

### Scoring for risk classification

**Figure 1:**
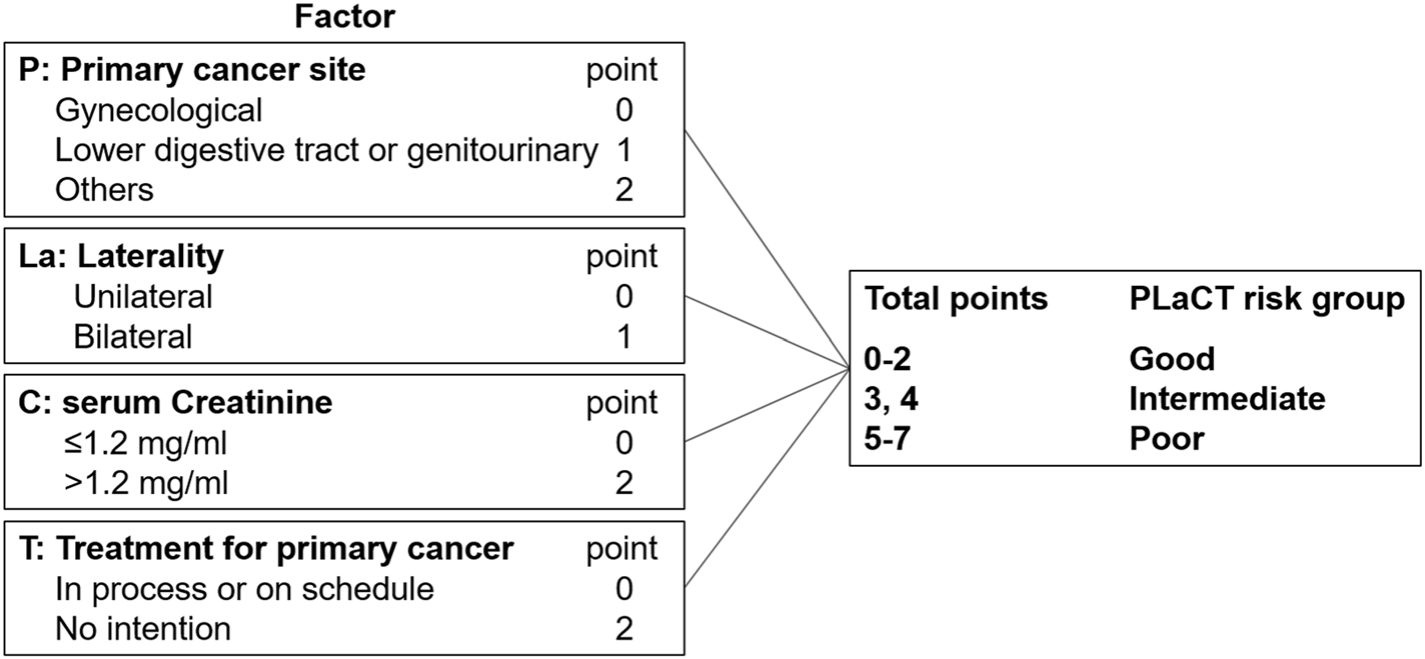
Scoring for PLaCT risk classification. Patients were divided into three risk groups (good, intermediate, and poor) based on the score calculated using four prognostic factors.

Patients were divided into three risk groups (good, intermediate, and poor) based on the score calculated using four prognostic factors (PLaCT: Primary cancer site, Laterality, sCr, and Treatment for primary cancer), and prospective follow-up was performed. These four factors were extracted in accordance with the results of our prior retrospective study^5^. Briefly, unfavorable prognostic factors of OS were identified by multivariate analysis using 61 MUO patients, and each factor was weighted on the basis of its *P* value. Regarding primary cancer site, GY cancer, lower digestive tract (LDT) and genitourinary (GU) cancers, and gastrointestinal (GI) and other cancers were given 0, 1, and 2 points, respectively. Additionally, bilateral MUO, sCr > 1.2 mg/dL, and no intention to treat primary cancer were given 1, 2, and 2 points, respectively. “On schedule” treatment for primary cancer at the time of scoring was given 0 points, even if the primary cancer would have received no treatment. If information regarding treatment for primary cancer was not available at the time of initial scoring, this factor was scored retrospectively at the time of final data collection. Therefore, all patients received a score from 0 to 7 in accordance with the above four prognostic factors. Patients with scores of 0–2, 3 or 4, and 5–7 were assigned to the good, intermediate, and poor risk group, respectively (Fig. 1).

### Endpoint

The primary endpoint was OS in each PLaCT risk group, whereas the secondary endpoint was stent failure-free survival (SFFS) in ureteral stent-indwelled patients. OS and SFFS were also analyzed in a cohort that excluded GU cancer patients because GU cancer causes intrinsic MUO; as treatment for cancer and MUO are often identical, the usefulness of the PLaCT risk classification score could be validated for both overall and extrinsic MUO. The date of enrollment was recorded as the start of observation. Dates of death were recorded in overall patients, whereas dates of ureteral stent failure were recorded in ureteral stent-indwelled patients. OS was measured from the start of observation to death from any cause. SFFS was measured from the start of observation to stent failure or death. We defined stent failure as the requirement for an alternative form of urinary diversion.

### Statistical analysis

Statistical analyses were performed using commercially available software GraphPad Prism 5 (GraphPad Software, San Diego, CA, USA). One-way analysis of variance and Student's t-test were used to assess differences among three groups and between two groups, respectively. The chi-squared test and chi-squared test for trend were used to analyze contingency tables. OS and SFFS were estimated using the Kaplan–Meier method; the log-rank test for trend was used to compare survival distributions. Statistical significance was defined as *P* < 0.05.

## Results

### Patient background and OS of overall patients

In total, 300 patients were enrolled in this study. The demographics of overall patients are shown in Table 1. The numbers of patients in the good, intermediate, and poor risk groups were 105, 106, and 89, respectively. Risk increased as patients became older. The proportion of women was highest in the good risk group. Risk increased with worse ECOG PS; risk also appeared to increase with the use of nephrostomy as management for MUO. There were significant differences in some hematological and blood biochemical measurements (hemoglobin, C-reactive protein, BUN, eGFR, total protein, and albumin) among risk groups. Although there were no differences in worst stenotic portion, the longest stenotic length was significantly greater in the poor risk group. With regard to PLaCT factors, there were significant differences in laterality, sCr, and treatment for primary cancer as a matter of course. Table 2 shows the distribution of primary cancer sites. The current study cohorts comprised 21 types of primary cancer sites and included five patients with cancer of unknown origin. The proportion of GY cancers decreased in the current study, whereas the proportion of other primary cancers in the current study was twice that of other primary cancers in the retrospective study^5^. Importantly, the number of malignant lymphomas increased to 26 in the current study. Each category of primary cancer sites comprised approximately 20% (minimum of 17% in GU and maximum of 22% in GY). The proportion of GY that was assigned 0 points was significantly greater in the good risk group, whereas the proportion of GI that was assigned 2 points was significantly greater in the poor risk group. Among all risk groups, there were no significant differences in the proportions of LDT and GU cancers that were assigned 1 point.

**Table 1:** Patient demographics of overall patients.

Variable	PLaCT risk group			*P* value
	Good	Intermediate	Poor	
Patients, *n* (%)	105 (35)	106 (35)	89 (30)	
**Median age (range), years**	65 (28–83)	68 (25–87)	71 (31–96)	0.0004
**Sex**, ***n*** **(%)**				
Male	23 (22)	66 (62)	37 (41)	0.0025
Female	82 (78)	40 (38)	52 (58)	
**ECOG PS,** ***n*** **(%)**				
0	68 (65)	33 (31)	18 (20)	< 0.0001
1	28 (27)	46 (43)	30 (34)	
2	3 (3)	14 (13)	12 (13)	
3	0 (0)	5 (5)	18 (20)	
4	2 (2)	3 (3)	8 (9)	
Unknown	4 (4)	5 (5)	3 (3)	
**Rescue of MUO,** ***n*** **(%)**				
Stenting	83 (79)	72 (68)	62 (70)	0.0001
Nephrostomy	3 (3)	22 (21)	21 (24)	
No	19 (18)	12 (11)	6 (7)	
**Worst stenotic portion**, ***n*** **(%)**				
Upper, upper-middle, and -lower	19 (18)	26 (25)	27 (30)	0.1188
Middle and middle-lower	26 (2)	30 (28)	27 (30)	
Lower	53 (50)	43 (41)	29 (33)	
Unknown	8 (8)	6 (6)	6 (7)	
**Median stenotic length (range), cm**	2.4 (0–14)	2.8 (0.4–15)	3.2 (0.5–20)	0.0045
**Median WBC count (range), × 10**^**3**^**/µL**	5.7 (0.5–20.2)	6.5 (1.5–20.3)	6.4 (2.4–24.8)	0.1372
**Median Hb (range), g/dL**	11.2 (6.6–16.0)	10.0 (5.5–14.5)	9.6 (5.7–14.3)	< 0.0001
**Median Plt count (range), × 104/µL**	23.4 (2.2–60.7)	23.9 (2.1–65.3)	22.1 (2.2–67.3)	0.7678
**Median CRP (range)**^**a**^**, mg/dL**	0.9 (0.0–26.8)	2.0 (0.0–16.5)	3.1 (0.0–39.0)	0.0097
**Median BUN (range)**^**a**^**, mg/dL**	15.0 (5.2–71.4)	23.9 (3.9–192)	36.1 (12.0–147)	< 0.0001
**Median eGFR (range)**^**a**^**, mL/min**	57.0 (6.0–146)	34.5 (1.0–123)	17.3 (2.5–110)	< 0.0001
**Median TP (range)**^**a**^**, g/dL**	6.7 (4.4–8.2)	6.3 (3.0–8.2)	6.1 (4.3–8.4)	0.0031
**Median Alb (range)**^**a**^**, g/dL**	3.6 (1.4–5.0)	3.3 (1.4–4.7)	3.2 (1.0–4.5)	< 0.0001
**Laterality,** ***n*** **(%)**				
Unilateral	92 (88)	56 (53)	13 (15)	< 0.0001
Bilateral	13 (12)	50 (47)	76 (85)	
**Median sCr (range), mg/mL**	0.84 (0.32–5.66)	1.44 (0.42–32.4)	2.78 (0.44–12.9)	< 0.0001
**Treatment for primary cancer,** ***n*** **(%)**				
Yes	104 (99)	86 (81)	21 (24)	< 0.0001
CT	69 (66)	72 (68)	16 (18)	
RT	2 (2)	4 (4)	1 (1)	
OP	9 (9)	1 (1)	1 (1)	
CT + RT	8 (8)	3 (3)	1 (1)	
CT + OP	16 (15)	5 (5)	1 (1)	
CT + RT + OP	0 (0)	1 (1)	1 (1)	
No	1 (1)	20 (19)	68 (76)	

*ECOG PS* Eastern Cooperative Oncology Group Performance Status, *MUO* malignant ureteral obstruction, *sCr* serum creatinine, *WBC* white blood cell, *Hb* hemoglobin, *Plt* platelet, *CRP* c-reactive protein, *BUN* blood urea nitrogen, *eGFR* estimated glomerular filtration rate, *TP* total protein, *Alb* albumin, *CT* chemotherapy, *RT* radiotherapy, *OP* operation.

^a^The numbers of missing data points are 23, 2, 2, 11, and 58 in CRP, BUN, eGFR, TP, and Alb, respectively.

**Table 2:** Distribution of primary cancer site.

Primary cancer site	Retrospective study cohort^a^	Overall current study cohort	PLaCT risk group			*P* value^b^
			Good	Intermediate	Poor	
Patients, *n* (%)	61	300 (100)	105 (35)	106 (35)	89 (30)	
**Gynecological**	21 (34)	66 (22)	40 (38)	13 (12)	13 (15)	< 0.0001
Ovary	7 (11)	32 (11)	21 (20)	8 (8)	3 (3)	
Cervix	13 (21)	25 (8)	14 (13)	4 (4)	7 (8)	
Uterine	1 (2)	9 (3)	5 (5)	1 (1)	3 (3)	
**Gastrointestinal**	13 (21)	62 (21)	11 (10)	20 (19)	31 (35)	< 0.0001
Stomach	12 (20)	58 (19)	11 (10)	19 (18)	28 (31)	
Esophagus	1 (2)	2 (1)	0 (0)	1 (1)	1 (1)	
GIST	0 (0)	2 (1)	0 (0)	0 (0)	2 (2)	
**Lower digestive tract**	10 (16)	59 (20)	24 (23)	20 (19)	15 (17)	0.2893
Colon	4 (7)	33 (11)	11 (10)	13 (12)	9 (10)	
Rectum	6 (10)	23 (8)	12 (11)	6 (6)	5 (6)	
Cecum	0 (0)	3 (1)	1 (1)	1 (1)	1 (1)	
**Genitourinary**	10 (16)	50 (17)	12 (11)	32 (30)	6 (7)	0.5198
Bladder	2 (3)	19 (6)	4 (4)	11 (10)	4 (4)	
Ureter	2 (3)	13 (4)	4 (4)	9 (8)	0 (0)	
Prostate	2 (3)	12 (4)	1 (1)	10 (9)	1 (1)	
Retroperitoneal sarcoma	3 (5)	3 (1)	1 (1)	2 (2)	0 (0)	
Adrenal gland	1 (2)	0 (0)	0 (0)	0 (0)	0 (0)	
Germ cell	0 (0)	2 (1)	1 (1)	0 (0)	1 (1)	
Urachus	0 (0)	1 (0)	1 (1)	0 (0)	0 (0)	
**Other**	7 (11)	63 (21)	18 (17)	21 (20)	24 (27)	0.0982
Malignant lymphoma	1 (2)	26 (9)	8 (8)	10 (9)	8 (9)	
Pancreas	2 (3)	12 (4)	5 (5)	3 (3)	4 (4)	
Breast	1 (2)	9 (3)	1 (1)	4 (4)	4 (4)	
Lung	2 (3)	6 (2)	1 (1)	2 (2)	3 (3)	
Liver	1 (2)	0 (0)	0 (0)	0 (0)	0 (0)	
Gallbladder	0 (0)	4 (1)	2 (2)	0 (0)	2 (2)	
Sarcoma	0 (0)	1 (0)	0 (0)	0 (0)	1 (1)	
Unknown origin	0 (0)	5 (2)	1 (1)	2 (2)	2 (2)	

*GIST* gastrointestinal stromal tumor.

^a^Reference^5^.

^b^Comparison among risk groups using chi-squared test for trend.

**Figure 2:**
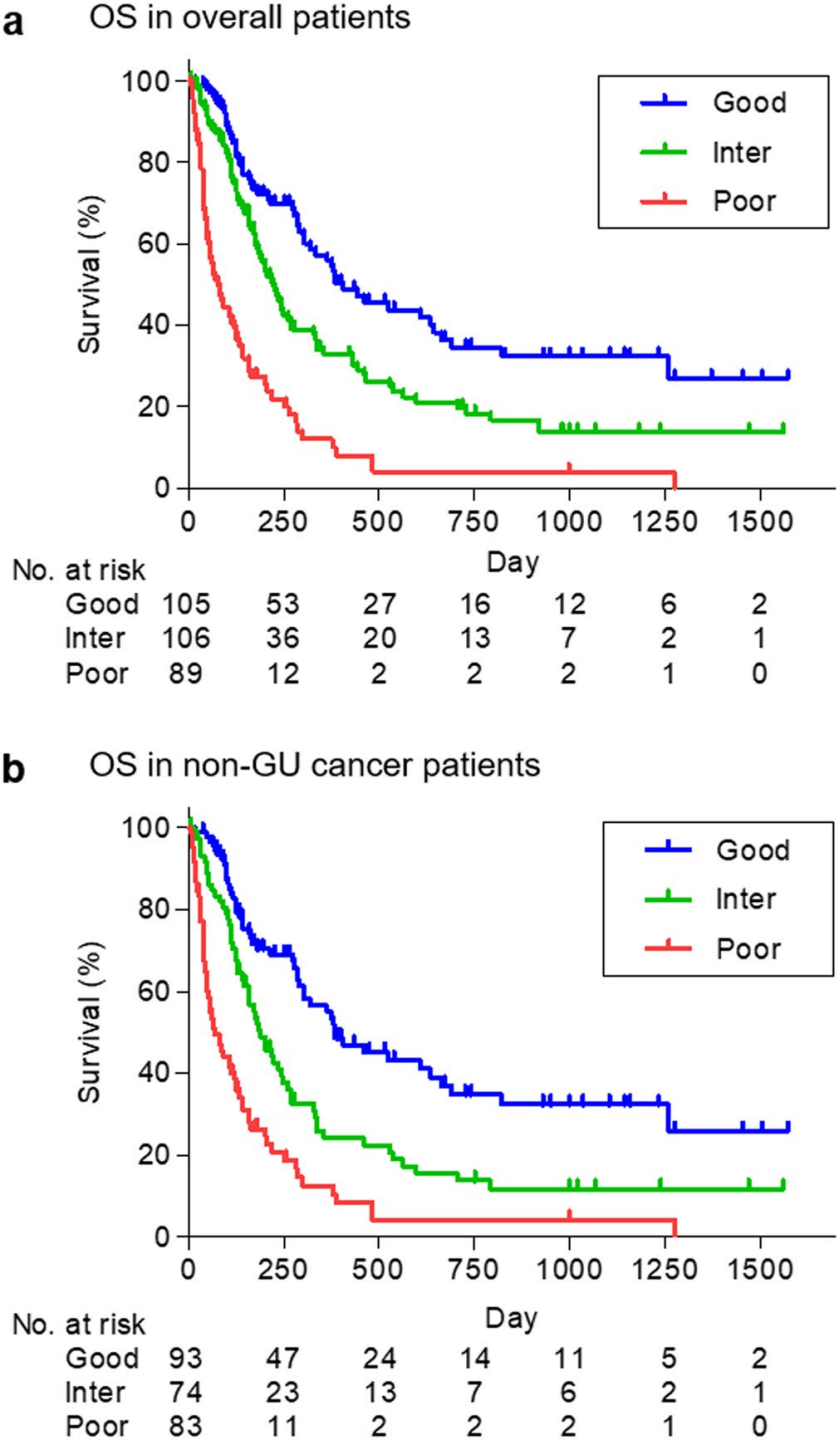
Overall survival of risk groups according to PLaCT risk classification. (**a**) Overall survival (OS) in overall patients (*P* < 0.0001). (**b**) OS in non-genitourinary (GU) cancer patients (*P* < 0.0001). Inter, intermediate. Statistical analyses were performed using GraphPad Prism 5 (https://www.graphpad.com/).

**Figure 3:**
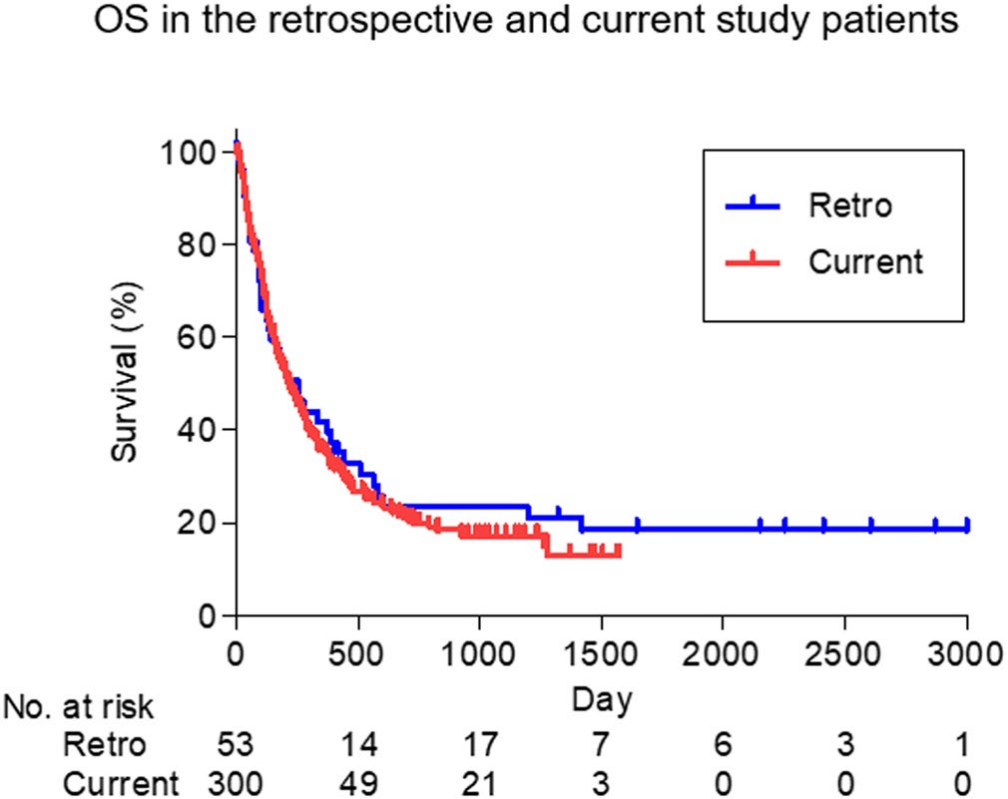
OS rates of overall patients in the current and retrospective studies. OS rates of overall patients in both studies were almost identical (*P* = 0.5429). Statistical analyses were performed using GraphPad Prism 5 (https://www.graphpad.com/).

Median survival times and 1 year survival rates of good, intermediate, and poor risk groups in overall patients were 406, 221, and 77 days, and 54.4%, 32.7%, and 8.0%, respectively (*P* < 0.0001, Fig. 2a). The numbers of patients with non-GU cancers in good, intermediate, and poor risk groups were 93, 74, and 83, respectively. Median survival times and 1 year survival rates of good, intermediate, and poor risk groups in patients with non-GU cancers were 383, 187, and 68 days, and 53.3%, 22.4%, and 10.5%, respectively (*P* < 0.0001, Fig. 2b). Notably, OS rates of overall patients were nearly identical in the current and retrospective studies (*P* = 0.5429, Fig. 3).

### Patient background and SFFS in ureteral stent-indwelled patients

Of the 300 patients, ureteral stents were indwelled in 217. The numbers of patients in the good, intermediate, and poor risk groups were 83, 72, and 62, respectively. There were significant differences in laterality, sCr, and treatment for primary cancer (Supplementary Table 1). The distribution of primary cancer sites is shown in Supplementary Table 2.

**Figure 4:**
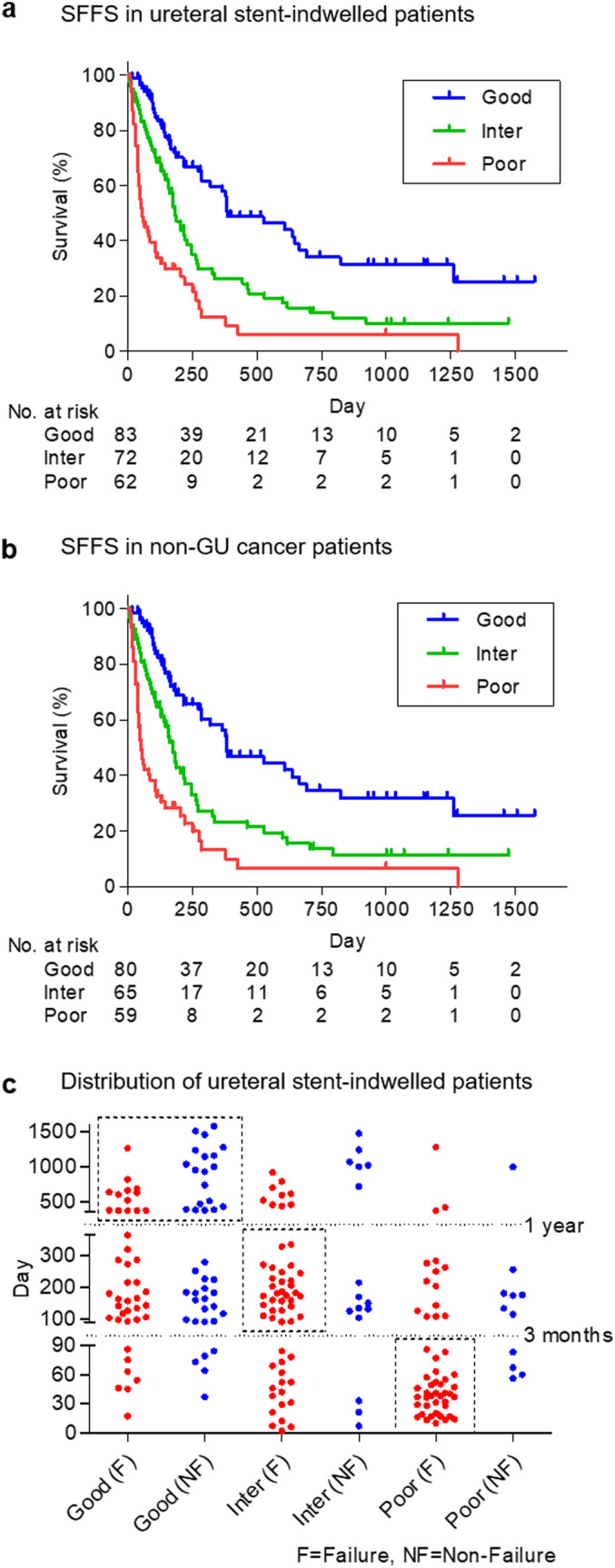
Stent failure-free survival of risk groups according to PLaCT risk classification. (**a**) Stent failure-free survival (SFFS) in overall patients (*P* < .0001). (**b**) SFFS in non-genitourinary (GU) cancer patients (*P* < 0.0001). (**c**) Distribution of SFFS in overall patients with boundaries of 3 months and 1 year. Although half of patients in the good risk group remained stent failure-free over 1 year (left upper square), most patients in the intermediate (Inter) and poor risk groups experienced stent failure between 3 months and 1 year (middle square), and within 3 months (right lower square), respectively. Statistical analyses were performed using GraphPad Prism 5 (https://www.graphpad.com/).

Median survival times and 1 year survival rates of good, intermediate, and poor risk groups in ureteral stent-indwelled patients were 385, 183, and 57 days, and 56.1%, 24.5%, and 9.3%, respectively (*P* < 0.0001, Fig. 4a). The numbers of patients with ureteral stent-indwelled non-GU cancers in good, intermediate, and poor risk groups were 80, 65, and 59, respectively. Median survival times and 1 year survival rates of good, intermediate, and poor risk groups in patients with ureteral stent-indwelled non-GU cancers were 383, 173, and 52 days, and 54.5%, 21.4%, and 9.9%, respectively (*P* < 0.0001, Fig. 4b). The number of stent failure events before death and median days to stent failure event in good, intermediate, and poor risk groups were 4, 9, and 8, and 201 (range 45–385), 73 (range 2–617), and 23 days (range 10–425), respectively. Notably, many patients in the good risk group had long stent patency; however, the largest population of patients in the intermediate risk group had stent failure including death between 3 months and 1 year, whereas the largest population of patients in the poor risk group had stent failure including death within 3 months (Fig. 4c).

## Discussion

This prospective multicenter study was performed to validate a novel risk classification score for OS that was previously introduced in our retrospective study of 61 patients with MUO^5^. Several risk classification scores for OS in advanced cancer patients with MUO have been reported thus far, and their constituent factors have shown considerable variation^6–9^. In addition, nearly all studies were retrospective, and their results have not been validated in prospective studies. Although a study of prospectively accumulated clinical and laboratory data from 208 patients suggested a risk classification score for OS, it has not been prospectively validated^8^. The current study of 300 advanced cancer patients with MUO reinforced the findings of our retrospective study. The inclusion of eight institutions in the study enabled the analysis of a variety of patients with MUO and controlled background noise in the data. In particular, the variety in primary cancer sites is important for establishment of the universal applicability of the risk classification score. The numbers of cancers of pancreas, breast, lung, gallbladder, and primary unknown origin were dramatically increased, as were the numbers of malignant lymphomas; this ensured that each category of primary cancer sites exhibited equal distribution. Moreover, the numbers of patients in each risk group were nearly equal, both in overall patients and in ureteral stent-indwelled patients, indicating that this approach functions effectively for classification.

There are several strengths in the PLaCT risk classification score. First, the PLaCT risk classification score can be applied to both extrinsic and intrinsic MUO. Because intrinsic MUO can largely be relieved by treatment of the primary cancer site, some studies of MUO have excluded intrinsic MUO^10^. The current study showed that all types of MUO, irrespective of primary cancer site, can be accurately classified by the PLaCT risk score based on the primary endpoint results. Moreover, even if patients with intrinsic MUO were removed from the cohort, 250 patients remained for analysis, and significant differences were found among the risk groups.

Second, the PLaCT risk classification score can be used to predict SFFS for ureteral stent-indwelled MUO patients. Ureteral stent placement is the most useful and frequently performed procedure for MUO; however, stent failure is one of the most challenging situations to manage with regard to MUO, because it is associated with kidney failure and symptomatic infection, as well as the need for emergent nephrostomy before progression of these conditions^11^. Patients in the good risk group are expected to maintain patency for > 1 year; thus, surgical dilation for ureteral stenosis may be useful for the management of MUO^12^. It remains unclear which procedures (e.g., ureteral stent or nephrostomy) should be primarily applied for MUO patients^13^. Nephrostomy as an initial treatment may be suggested for patients in the poor risk group because the proportion of stent failure among these patients was high and the survival time was < 3 months^14^. The use of a metallic ureteral stent may be appropriate for patients in the intermediate risk, who showed OS between 3 months and 1 year, because this type of stent can maintain strong patency up to 1 year without exchange^15^.

Finally, the PLaCT risk classification score is both simple and appropriate and can be calculated easily using four factors that can be collected rapidly. Some reports of risk factors and classification for MUO are particularly complicated with regard to prediction of OS^16^. Conversely, some systems might be too simple to accurately classify all MUO patients over a wide range of backgrounds^8^. Similar OS findings in our prior retrospective study and the current prospective study indicate that the PLaCT risk classification score is reliable^5^. Physicians who are engaged in cancer treatment can calculate the score and classify MUO patients into three PLaCT risk groups without requiring aid from urologists. PLaCT risk classification may be helpful to consider treatment strategy for MUO.

Despite several strengths, the current study had a number of limitations. Sample size may be insufficient to determine precise statistical significance. All patients were of Japanese ethnicity, and the distribution of cancers may differ in patients from other ethnic backgrounds. The treatment for MUO was determined at discretion of urologists in charge, and this might cause the biased distribution of treatment. Although we focused on patients with MUO regardless of their clinical stages, who are usually regarded as advanced cancer patients, this study lacks the staging information. The correct staging information may contribute to better classification for OS. The extended duration of study enrollment may have distorted survival time because cancer treatment has rapidly progressed in recent years. In particular, immune checkpoint inhibitors may have affected survival in several types of cancers. A variety of treatment for primary cancer in the good risk group may indicate cure potential and the better clinical stage, contributing to the better OS. Factors previously reported as significant with regard to OS or SFFS were not considered as prognostic variables in the current study because it was conducted to validate the findings of our previous retrospective study. Because many variables, such as age, gender, PS, Hb, and so on, showed statistical significance among risk groups in the current study, a novel risk classification score that predicts OS more accurately than the PLaCT risk classification score may be developed from the data in the current study.

The current study was performed as a prospective multicenter study to validate a risk classification score for OS in advanced cancer patients with MUO for the first time. The PLaCT risk classification score can be concisely calculated and used to divide MUO patients into three risk groups with distinct survival times. This classification score will aid in establishing prognosis and treatment strategy for all physicians engaged in cancer treatment and assist urologists in the selection of suitable procedures to manage MUO.

## Supplementary Information

Supplementary Tables.

## Data Availability

All data generated or analysed during this study are included in this published article (and its Supplementary Information files).
