# Supplementary material for: A novel risk classification score for malignant ureteral obstruction: a multicenter prospective validation study
Source: Sci Rep. 2021 Feb 24;11:4455. doi: 10.1038/s41598-021-84054-7 (PMC7904864; doi:10.1038/s41598-021-84054-7)
Supplement: Supplementary file 1 — Supplementary Tables. [file 41598_2021_84054_MOESM1_ESM.pdf]

A novel risk classification score for malignant ureteral obstruction: a multicenter prospective validation study

Kouji Izumi [a,*], Takashi Shima [b], Kazuyoshi Shigehara [a,c], Kiyoshi Sawada [d], Renato Naito [a,e], Yuki Kato [a,f], Mitsuo Ofude [c,g], Hiroshi Kano [a,h], Hiroaki Iwamoto [a], Hiroshi Yaegashi [a,c], Kazufumi Nakashima [a], Masashi Iijima [a], Shohei Kawaguchi [a,b], Takahiro Nohara [a,b], Yoshifumi Kadono [a], Atsushi Mizokami [a]

[a]Department of Integrative Cancer Therapy and Urology, Kanazawa University Graduate School of Medical Science, Kanazawa, Japan

[b]Department of Urology, Toyama Prefectural Central Hospital, Toyama, Japan

[c]Department of Urology, Ishikawa Prefectural Central Hospital, Kanazawa, Japan

[d]Department of Urology, Municipal Tsuruga Hospital, Tsuruga, Japan

[e]Department of Urology, Komatsu Municipal Hospital, Komatsu, Japan

[f]Department of Urology, Fukui-ken Saiseikai Hospital, Fukui, Japan

[g]Department of Urology, Kouseiren Takaoka Hospital, Takaoka, Japan

[h]Department of Urology, National Hospital Organization Kanazawa Medical Center, Kanazawa, Japan

Supplementary Table 1. Patient demographics of ureteral stent-indwelled patients				
	PLaCT risk group			
Variable	Good	Intermediate	Poor	*p* value
Patients, *n* (%)	83 (38)	72 (33)	62 (29)	
Median age (range), y	64 (28–83)	66 (25–87)	72 (31–95)	0.0005
Sex, *n* (%)				
Male	15 (18)	43 (60)	24 (39)	0.0035
Female	68 (82)	29 (40)	38 (61)	
ECOG PS, *n* (%)				
0	52 (63)	27 (38)	13 (21)	< 0.0001
1	24 (29)	26 (36)	24 (39)	
2	1 (1)	11 (15)	8 (13)	
3	0 (0)	3 (4)	11 (18)	
4	2 (2)	1 (1)	3 (5)	
Unknown	4 (5)	4 (6)	3 (5)	
Laterality, *n* (%)				
Unilateral	92 (88)	56 (53)	13 (15)	< 0.0001
Bilateral	13 (12)	50 (47)	76 (85)	
Median sCr (range), mg/mL	0.83 (0.32–5.66)	1.46 (0.42–32.4)	2.59 (0.96–12.9)	< 0.0001
Treatment for primary cancer, *n* (%)				
Yes	82 (99)	59 (82)	19 (31)	< 0.0001
No	1 (1)	13 (18)	43 (69)	
Worst stenotic portion, *n* (%)				
Upper, Upper-Middle, and Upper-Lower	17 (20)	21 (29)	21 (34)	0.0259
Middle and Middle-Lower	21 (25)	27 (38)	22 (35)	
Lower	38 (46)	19 (26)	15 (24)	
Unknown	7 (8)	5 (7)	4 (6)	
Median stenotic length (range), cm	2.4 (1.1–12)	3.0 (0.4–15)	3.9 (0.5–20)	0.0032
Median WBC count (range), $\times 10^3/\mu L$	5.6 (0.5–20.2)	6.2 (1.5–20.3)	6.0 (2.4–24.8)	0.2275
Median Hb (range), g/dL	11.0 (6.6–16.0)	10.2 (6.0–14.5)	9.8 (5.7–13.9)	0.0007
Median Plt count (range), $\times 10^4/\mu L$	22.9 (2.2–60.7)	22.1 (2.1–62.0)	22.0 (2.2–67.3)	0.8344
Median CRP (range)[a], mg/dL	1.3 (0.0–26.8)	1.6 (0.0–16.5)	2.9 (0.0–39.1)	0.1003
Median BUN (range)[a], mg/dL	14.5 (5.2–71.4)	23.0 (5.8–192)	35.4 (14.5–144)	< 0.0001
Median eGFR (range)[a], mL/min	56.0 (6.0–146)	36.5 (1.0–124)	18.0 (2.5–54.0)	< 0.0001
Median TP (range)[a], g/dL	6.6 (4.4–8.2)	6.3 (3.0–8.2)	6.2 (4.3–8.1)	0.0176

Median Alb (range)[a], g/dL	3.6 (1.4–5.0)	3.3 (1.4–4.7)	3.2 (1.0–4.4)	0.0025

Abbreviations: ECOG PS, Eastern Cooperative Oncology Group Performance Status; MUO, malignant ureteral obstruction; sCr, serum creatinine; WBC, white blood cell; Hb, hemoglobin; Plt, platelet; CRP, c-reactive protein; BUN, blood urea nitrogen; eGFR, estimated glomerular filtration rate; TP, total protein; Alb, albumin. [a]The numbers of missing data points are 14, 2, 2, 7, and 42 in CRP, BUN, eGFR, TP, and Alb, respectively.

Supplementary Table 2. Primary cancer sites of ureteral stent-indwelled patients

Primary cancer site	Overall current study cohort	PLaCT risk group			
		Good	Intermediate	Poor	*p* value[a]
Patients, *n* (%)	217 (100)	83 (38)	72 (33)	62 (29)	
Gynecological	52 (24)	36 (43)	10 (14)	6 (10)	< 0.0001
Ovary	27 (12)	19 (23)	6 (8)	2 (3)	
Cervix	16 (7)	12 (14)	3 (4)	1 (2)	
Uterine	9 (4)	5 (6)	1 (1)	3 (5)	
Gastrointestinal	52 (24)	10 (12)	17 (24)	25 (40)	< 0.0001
Stomach	49 (23)	10 (12)	16 (22)	23 (37)	
Esophagus	2 (1)	0 (0)	1 (1)	1 (2)	
GIST	1 (0)	0 (0)	0 (0)	1 (2)	
Lower digestive tract	46 (21)	20 (24)	17 (24)	9 (15)	0.1803
Colon	27 (12)	8 (10)	12 (17)	7 (11)	
Rectum	17 (8)	11 (13)	4 (6)	2 (3)	
Cecum	2 (1)	1 (1)	1 (1)	0 (0)	
Genitourinary	16 (7)	4 (5)	9 (13)	3 (5)	0.8607
Bladder	4 (2)	1 (1)	2 (3)	1 (2)	
Ureter	4 (2)	0 (0)	4 (6)	0 (0)	
Prostate	2 (1)	0 (0)	1 (1)	1 (2)	
Retroperitoneal sarcoma	3 (1)	1 (1)	2 (3)	0 (0)	
Adrenal gland	0 (0)	0 (0)	0 (0)	0 (0)	
Germ cell	2 (1)	1 (1)	0 (0)	1 (2)	
Urachus	1 (0)	1 (1)	0 (0)	0 (0)	
Other	51 (24)	13 (16)	19 (26)	19 (31)	0.031
Malignant lymphoma	22 (10)	6 (7)	9 (13)	7 (11)	
Pancreas	10 (5)	4 (5)	3 (4)	3 (5)	
Breast	7 (3)	1 (1)	3 (4)	3 (5)	
Lung	5 (2)	0 (0)	2 (3)	3 (5)	
Liver	0 (0)	0 (0)	0 (0)	0 (0)	
Gallbladder	3 (1)	2 (2)	0 (0)	1 (2)	
Sarcoma	0 (0)	0 (0)	0 (0)	0 (0)	
Unknown origin	4 (2)	0 (0)	2 (3)	2 (3)	

Abbreviation: GIST, gastrointestinal stromal tumor. [a]Comparison among risk groups using chi-squared test for trend.
